# An attachment theoretical perspective for the neural representation of close others

**DOI:** 10.1093/scan/nsz010

**Published:** 2019-02-01

**Authors:** Anne C Laurita, Cindy Hazan, R Nathan Spreng

**Affiliations:** 1 Department of Human Development, Cornell University, Ithaca, NY, USA; 2 Health Promotion & Prevention Services, University Health Services, Princeton University, Princeton, NJ, USA; 3 Laboratory of Brain and Cognition, Montreal Neurological Institute, Department of Neurology and Neurosurgery, McGill University, Quebec, Canada; 4 Departments of Psychiatry and Psychology, McGill University, Quebec, Canada

**Keywords:** adult attachment, close relationships, fMRI

## Abstract

Recent investigations in neuroscience elucidate the neural basis of close other cognitive representations, which serve functions central to our health and happiness. Yet, there are persistent barriers to this research, including disparate research methods and the absence of a common theoretical background. The present review connects neuroimaging and attachment theory within a novel social, cognitive and affective framework. We apply attachment theory to understand why we would expect cognitive representations of close others to be different from other social neural representations. Developing reliable markers of attachment is a critical step in mapping close other neural representations. We then examine existing neuroimaging literature on close other representations, highlighting the recruitment of neural systems supporting reward, motivation and distress alleviation, in addition to the mirror neuron system, default network and salience network. We then review the methodologies of past studies, revealing a diverse array of self-report measures assessing `closeness’ and social cognitive tasks that, taken together, preclude meaningful synthesis of findings. Lastly, we discuss specific behavioral measures of attachment and closeness with recommendations for the field. This attachment framework integrates brain and behavioral sciences and unites theoretical principles with empirical methods to further our understanding of how the brain represents close others.

I love you without knowing how, or when, or from where,

I love you straightforwardly, without complexities or pride;

so I love you because I know no other way

than this: where *I* does not exist, nor *you*,

so close that your hand on my chest is my hand,

so close that your eyes close as I fall asleep.

-Pablo Neruda, One Hundred Love Sonnets: XVII, 1959;

Translated by Stephen Tapscott

## Introduction

If you are able to suspend disbelief for a moment, assume that two individuals’ bodies could so precisely synchronize as a function of mere affection. Then perhaps Neruda’s prose does accurately reflect feelings of intense love. His imagery evokes a level of intimacy and connection reserved only for certain close relationships. Humankind’s fascination with the experience of love and thinking about loved-ones stretches far back through history, long before Neruda wrote his sonnets. This curiosity flourishes today, too; the observation of biobehavioral synchrony, or the sensitization to and mirroring of another’s physiological and behavioral cues, in pair bonds (see Feldman, 2017, for review), for instance, provides intriguing support for this particular poetic musing. Across scientific domains we are relatively new to the formal exploration of close social bonds. Yet, we have already made great strides in amassing evidence for these unique attachment relationships that are operative across the lifespan and manifest in affect, behavior, cognition and physiology.

From an ethological perspective, interest in investigating close relationships stems from knowledge of the social pressures unique to the human species, such as navigation of complex social hierarchies and formation of successful mating relationships (see Fletcher *et al*., 2015, for review). Our ability to cognitively represent others and respond accordingly differentiates us from our primate relatives (Tomasello, 1999). Evolutionary theorists postulated the social brain hypothesis to account for humans’ unique social-cognitive skills. As humans evolved, living in group settings, individuals had to manage increasing complexity and number of social relationships. This evolutionary pressure was associated with markedly larger brains (Dunbar, 1998). The capacity to successfully navigate through personal interactions remains crucial for human survival. We understand that the formation and maintenance of close relationships is essential for defining one’s sense of self (Vygotsky, 1978), surviving to mate and raise young (Buss and Schmitt, 1993) and bolstering physical and mental health throughout the lifespan (House *et al*., 1988; Kiecolt-Glaser and Newton, 2001; Cohen, 2004; Cornwell and Waite, 2009; Sbarra and Coan, 2018). The ability to stratify personal relationships—differentiating close from other more distal connections across social networks—is especially important for successful social navigation.

Social-cognitive neuroscientists have begun to map this `social landscape’ to the complex architecture of the brain. Patterns of neural activity have been identified that differentiate cognitive representations of close others from less-close others (Krienen *et al*., 2010; Laurita *et al*., 2017). Yet, efforts to integrate social psychology and neuroscience research to define how one neurally represents close others have proven challenging; there are several persistent barriers, including the absence of a common theoretical framework, disparate research methods and measurement challenges. In order to use measures of brain activity to make inferences about close other representations in the brain, the emerging field of close relationship neuroscience needs to make progress toward clearer measurements and evaluation of constructs defining close relationships. The present review connects past work, providing a common social neuroscientific framework for understanding representations of our closest social relationship partners: attachment figures. We aim to show how clear integration of a few, central concepts across the relevant bodies of literature provides a novel perspective for the emergent field of close relationship neuroscience, elucidating a path for future inquiry.

## Outline and scope of the present review

The primary objective of this review is to provide an integrated common framework for understanding the neural representation of our closest social relationships. Central to this framework is the notion of reciprocity between social neuroscience and attachment theory; social neuroscience can provide an organizational structure for understanding the neural representation of close social relationships, including attachment relationships, and attachment theory, in turn, can provide behavioral and cognitive markers to guide social neuroscientific investigations. We begin with a survey of the theoretical and social psychological bases of attachment, examining how they might inform social neuroscience research. We emphasize the potential for attachment theory to guide functional neuroimaging investigations of specific close other mental representations. The hallmark of attachment bonds is the feeling of security associated with attachment figures’ availability. Attachment theory suggests that behavior towards a certain close other differs fundamentally from behavior towards more distant others, signaling that neural representations of social others may be similarly stratified across a proximal-distal continuum. Utilizing criteria provided by attachment theory—for example, by stratifying social relationships by self-report of felt security—could provide necessary guidance in differentiating the corresponding neural representations. Attachment theory can provide neuroscientists with specific behavioral and cognitive constructs to examine.

We next present recent and seminal social cognitive neuroscience studies that shed light on the highly relevant domains of reward responses to close relationship partners, social emotion regulation and social information processing. Collectively, this work has implicated a wide array of brain regions in representing close relationships including the following: the dopaminergic and opioid reward systems; the limbic system in emotional regulation and distress alleviation; the mirror neuron system (MNS); the default network, responsible for internally-directed thought and social cognition; and the salience network in differentiation of unique close other representations. The studies we review adopted diverse approaches to operationalize and measure the construct of close other representations. In the next section of our review, we evaluate these approaches. We discuss core methodological challenges in this area, including the following: use of terminology relating to close relationships and to attachment bonds, a smaller subset of those relationships examined; assessment of relationship length; use of self-report measures of relationship closeness and quality; differing neuroimaging task paradigms; and the potential impact of demographic factors such as sex, sexual orientation and age. Of particular interest are experimental parameters reflecting how researchers measure `closeness‘ of close others in relation to neural responses. Such methodological discrepancies preclude meaningful integration of findings across concepts and fields. To conclude, we offer a series of recommendations designed to promote an interdisciplinary approach for mapping the neural representation of unique close relationships, conceptualized as attachment bonds. We review behavioral methods to assess close adult relationships, including attachment bonds, and with recommendations for the field. Developing reliable markers of attachment, grounded in social psychological theory, is a critical step in mapping close relationship representations in the human brain.

As this inquiry comes at a critical time for close relationship neuroscience (see Feldman, 2017, for review; Laurita *et al*., 2017), we seek to situate this work within a broader context of the disparate fields upon which it draws. First, we focus this review solely on close relationships in adulthood, gathering evidence primarily from studies of adult romantic relationships and secondarily from studies of parent/adult–child relationships or close adult friendships. We do not review studies of infants’ or children’s close relationships, nor do we review studies of clinical populations, adult or otherwise. Second, we utilize attachment theory as a normative, quantitative framework for our discussion. Motivated by Bowlby’s (1973) traditional model, our perspective hinges on the quantifiable presence (*vs* non-presence) of attachment to characterize close relationships. We do not approach attachment theory from Ainsworth *et al*.’s (1978) tradition of observing individual differences in attachment style; researchers have yet to integrate relationship-observational methods with collection of neuroimaging data, and we do not yet have sufficient data to delve into these individual differences. Third, we use specific language, discussing `close other representations’ as a unifying construct throughout our review of results and methods from existing research. Towards the end of this work, we apply the theoretical framework of attachment and incorporate references, where useful, to `adult attachment figure representations’, concretely illustrating the boundaries of our framework that ties together social neuroscience and attachment theory. We avoid appropriating terminology from clinical or developmental domains, whose language is too often conflated with that from other related disciplines. Fourth, we draw exclusively from the body of work using blood oxygen level-dependent (BOLD) functional magnetic neuroimaging (fMRI). Most existing research on the neuroscience of human social relationships has used this method to gain spatial and temporal information about brain function in response to social stimuli. Within this coarse methodological focus, we parse out different results by calling attention to differing backgrounds and smaller methodological choices, such as specific fMRI tasks and contrasts used. We made these choices to narrow our focus and to avoid confusing concepts or findings from traditionally disparate areas of research.

We conclude by proposing a standardized battery of relationship measures, consistent with the tenets of attachment theory. We argue that standardized assessments, including measures of attachment status, style and relationship quality, are necessary to develop comprehensive, reliable and replicable representations of real-world attachment bonds.

## Utility of the adult attachment framework

Within the attachment literature—across studies of infants’ primary caregivers and adults’ romantic partners—one hallmark of these unique, close social bonds is the feeling of security, and concomitant affect-regulatory benefits, associated with attachment figures’ presence (Bowlby, 1973; Sroufe and Waters, 1977; Mikulincer and Shaver, 2007). Bowlby (1973) initially theorized that the function of attachment for infants was to support maintenance of proximity with a primary caregiver. Lack of perceived proximity and the accompanying distress activates this system, whereas comfort and the ability to explore are achieved through this system of attachment behavioral dynamics (Bowlby, 1973). These relationships are therefore characterized by four behavioral `features‘: proximity seeking, separation distress, safe haven and secure base. Individuals direct the behaviors of seeking and maintaining physical nearness to (proximity seeking); experiencing distress when separated from (separation distress); seeking comfort from when distressed (safe haven); and utilizing their continued support to further explore the surrounding environment (secure base) toward attachment figures (Mikulincer and Shaver, 2007). Observational studies of young children were the first to demonstrate the important role of attachment figures in pacifying separation-related distress upon reunion (Ainsworth *et al*., 1978). Ainsworth’s work paved the way for a research tradition of examining individual differences in patterns of behavioral response, observed and noted as `attachment styles’.

Attachment theory has since been extended to explain certain close, romantic relationships in adulthood (Hazan and Shaver, 1987). The overarching adult attachment framework serves as a predominant paradigm for understanding the regulatory powers of our closest social bonds (Hazan *et al*., 2004; Pietromonaco *et al*., 2006) and the long-term psychological and physiological health benefits conferred by these relationships (e.g. Beck *et al*., 2013; Sbarra and Coan, 2018). However, physical proximity is not always needed for felt security; once one has a mental representation of an attachment figure (also known as an internal working model), he or she is inherently shaping expectations, behaviors and utilization of this system on that cognitive representation. The so-called `chronic accessibility‘ of attachment figure mental representations (Andersen and Cole, 1990; Baldwin *et al*., 1996)—by which these representations are not just rich and detailed in content but also quick to be recalled and utilized—comes about due to learning and conditioning under this inborn system of attachment bonding, operative across the lifespan. Recent work indicates that there are also specific neural signatures of chronic accessibility, namely decreased activation in several key regions of interest, when thinking of an attachment figure parent or child (Laurita *et al*., 2018).

Since cognitive representations of attachment figures are thought be chronically accessible and relevant for emotion-regulation, they are inherently different in content and utilization from representations of others—acquaintances, friends or even ourselves (Pietromonaco *et al*., 2006; Mikulincer and Shaver, 2007). Mental representations of close others in adulthood are composed of highly salient social memories and, often, function independent of context. In early development, parents serve as our primary attachment figures; in young adulthood and beyond, romantic partners will often serve this role (Hazan *et al*., 1991; Hazan and Zeifman, 1999; Nickerson and Nagle, 2005). Repeated utilization of romantic partner mental representations is important for the maintenance of long-term, mutually-beneficial pair bonds (Mikulincer and Shaver, 2007).

Within the context of pair bonds, romantic partner mental representations have been further conceptualized as cognitive expansions of the self (Aron and Aron, 1986; Coan and Sbarra, 2015). Cognitive representations of attachment figures play a role in the pursuit of partner-specific interpersonal goals (Fitzsimons and Bargh, 2003) and in intertwining the cognitive and emotional contexts of both relationship partners (Zayas *et al.*, 2002). Moreover, these representations can influence our perceptions of, and responses to, others in our social world through a process known as social-cognitive transference (Andersen and Cole, 1990). Experimental studies (e.g. Günaydin *et al*., 2012) demonstrate the occurrence of social-cognitive transference, in which internal working models of close others can actually influence how novel social stimuli are perceived and encoded. The theory of social-cognitive transference proposes that mental representations of attachment figures strongly influence how we judge others in our social world (Andersen and Cole, 1990; Günaydin *et al*., 2012).

Beyond these effects, of great importance are the ramifications of attachment figure mental representation utilization in the face of stressors—at the affective, behavioral, neural and cognitive levels. Recent research demonstrates that attachment figure mental representations serve various functions contributing to our health and happiness. Just bringing to mind the cognitive representation of one’s romantic partner can promote recovery from recalling upsetting autobiographical memories (Selcuk *et al*., 2012), provide distress alleviation when giving a public speech (Grewen *et al*., 2003), decrease the neural response to threat with partner hand-holding (Coan *et al*., 2006) and reduce the subjective experience of pain, even above one’s described pain threshold (Eisenberger *et al*., 2011). Importantly, we have evidence to support the notion that mental representations are not immutable; representations themselves can be altered, at the levels of cognition, behavior and the brain, based on felt security (Collins and Feeney, 2004).

Both theoretical and empirical work support the uniqueness of attachment figure representations, especially within the context of romantic relationships. In many cases, the presence of intrinsically rewarding contact comfort and sexual activity indicate that romantic partnerships are uniquely intimate attachment bonds by nature (Zayas *et al*., 2015). As an attachment bond with a romantic partner forms, this individual becomes integrated into one’s cognitive sense of self (Aron and Aron, 1986) and influences one’s physiological homeostatic functions (Pietromonaco *et al*., 2013). Biobehavioral synchrony, or physiological co-regulation, is often present such pair bonds (for review, see Feldman, 2017).

Because of the powerful role of attachment figure mental representations in forming and maintaining close bonds and, more broadly, in assisting individuals with navigation of their social environments, it is likely that these representations have unique neural signatures. Neural representations of attachment figures can be understood, on a fundamental level, like all other social representations; they are distributed across multiple networks in the brain and may recruit different neural regions depending on the context in which they are utilized. Yet, hypotheses directly reflective of the features of attachment discussed above would enable examination of the uniqueness of these representations. Attachment has a rich theoretical and empirical literature, and rigorous studies of its behavioral dynamics can provide a foundational understanding onto which social, neural processes may begin to be mapped, without reducing either field (see Krakauer *et al*., 2017 for a recent discussion of this approach as applied across other neuroscientific domains). We propose that, by utilizing adult attachment criteria, researchers can implement theoretically-driven empirical studies and finer-grained corresponding analyses to differentiate our closest of social relationships representations in the brain. If we apply adult attachment to derive more precise operational definitions of close relationships, we can begin to disentangle the important functional regions and networks of the brain we predict to be recruited in such social cognitive processing.

## Diverse findings from fMRI studies of close other representations

Close other neural representations have been approached from two historically distinct but increasingly overlapping fields of thought through use of functional neuroimaging (fMRI) methods. Accordingly, results show diversity in the brain regions and networks, or large-scale systems of functionally connected brain regions, implicated in creating, updating and utilizing these mental representations. Social and affective neuroscientific studies focus on motivation and reward conditioning to close others and the affect-regulatory capabilities close others impart. These investigations consistently implicate the recruitment of reward and distress-alleviation systems in the brain (e.g. Bartels and Zeki, 2000; Xu *et al*., 2011; Acevedo *et al*., 2012). Cognitive neuroscientific investigations, on the other hand, focus on characterizing the differential cognitive representations of social others and of social distance. These studies typically investigate how close other representations are created over time through the encoding and retrieval of personal information and the accumulation of social memories. This body of work examines how the brain supports and updates representations of close social others and relates them to representations of the self, repeatedly demonstrating roles for neural systems involved in memory and internally-directed thought (e.g. Heatherton *et al*., 2006, Krienen *et al*., 2010; Wang *et al*., 2012). The different theoretical and methodological approaches likely result in the varied findings across individual studies of close others neural representations. In the following section, we review existing findings, highlighting several specific studies that have most meaningfully, as evidenced by citations, contributed to our emerging understanding of the neural representation of close others.

### Limbic system: reward pathways and emotion regulation

Limbic system activity plays a critical role in close other mental representations. The mesocorticolimbic and nigrostriatal dopaminergic reward pathways are involved in motivating attachment bond formation and maintenance, by way of conditioning to the presence of a romantic relationship partner (Fisher *et al*., 2005). Attachment figure representations become imbued with high reward as positive experiences accumulate with these individuals. Behaviorally, this system manifests as a cycle of attachment features; we seek proximity to those who provide us with a secure base. Existing social neuroscientific literature provides ample support for this facet of close other neural representations, finding recruitment of brain regions such as the ventral tegmental area (VTA), ventral and dorsal striatum, mid-insula, caudate head and putamen (Bartels and Zeki, 2000; Aron *et al*., 2005; Zeki and Romaya, 2010; Stoessel *et al*., 2011; Xu *et al*., 2011; Acevedo *et al*., 2012; Inagaki and Eisenberger, 2012; Xu *et al*., 2012; Scheele *et al*., 2013; Langeslag *et al*., 2014; Inagaki *et al*., 2015; Inagaki *et al*., 2016).

#### Reward responses to romantic partners: a neurochemical impetus for proximity seeking

The topic of early-stage, pre-attachment romantic relationships initially attracted social neuroscientists utilizing BOLD fMRI. Early-stage relationships are associated with feelings of euphoria and heightened neurochemical reward (Aron *et al*., 2005), and individuals in these relationships seek proximity to their partners who provide such reward. Several of the studies above examine early-stage, intense relationships characterized by feelings of infatuation. In the earliest assessment of the neural basis of romantic love, participants who reported being deeply `in love‘ were instructed to look at photographs of their romantic partners and of three different friends while in the fMRI scanner (Bartels and Zeki, 2000). Results showed increased activation in medial insula, caudate nucleus, putamen and anterior cingulate cortex (ACC) when participants looked at their romantic partners’ photos. These neural regions are dopamine-rich and are consistently recruited in reward paradigms. This activation pattern was investigated in another sample of participants in early-stage, intense romantic relationships and increased activations specific to romantic partners were again found in dopamine-rich areas of the brain such as right VTA and medial caudate nucleus (Aron *et al*., 2005). Individuals `happily in love’ in early-stage romantic relationships recruit bilateral insula and ACC more often than those recently separated from a romantic partner (Stoessel *et al*., 2011). Study participants from an Eastern culture, too, recruit VTA and caudate in representing early-stage romantic partners (Xu *et al*., 2011). Directing attention toward a beloved, early-stage romantic partner *vs* a friend has also been associated with increased ventral striatum activity (Langeslag *et al*., 2014).

The past several years have seen a shift in focus reflecting growing interest in the neuroscience of attachment, under the broader umbrella of social neuroscience research; recent studies have examined the role of motivation and reward systems in stable, longer-term adult romantic relationships. In 2012, the first and only longitudinal study on this topic examined the progression from early-stage passionate love to longer-term romantic relationships (Xu *et al*., 2012). Participants included individuals who, at 40-month follow-up, were together with their romantic partners from the first assessment and others who had since broken up. Results showed that partner-related activity in the tail of the caudate during the early-stage assessment was associated with remaining together 40 months later, as well as with higher self-reported commitment to the relationship. A second, somewhat counterintuitive set of activational effects also emerged, wherein lower early-stage activity in medial orbitofrontal cortex and nucleus accumbens (NAcc) was associated with greater commitment, happiness and longevity of participants’ relationships at 40-month follow-up. The directionality of Xu *et al*.’s (2012) second set of activational effects was brought into question by another foundational study of long-term relationship representations. Acevedo *et al*. (2012) made an important advance, examining neural representations of long-term romantic partners, using a photo-viewing paradigm. They found that individuals who reported high, passionate love for a long-term spouse showed significant patterns of neural activation in response to partner images *vs* acquaintance images in the VTA and substantia nigra. Furthermore, the authors found that greater closeness—measured by one specific social-cognitive measure—was related to greater VTA activity in response to partner images *vs* friend images, suggesting that long-term relationships are also inherently rewarding. Relationship length (here, years married) was positively correlated with activation of NAcc and caudate in response to romantic partner *vs* friend. There is also evidence that this pattern of findings holds across samples of non-heterosexual individuals in long-term romantic relationships (Zeki and Romaya, 2010).

Other recent research adds breadth to our understanding of the neural reward system’s role in representing close others. The neuropeptides oxytocin and vasopressin also interact with dopamine in neural reward processing (Love *et al*., 2012) and support long-term pair bond formation (e.g. Grewen *et al*., 2005; Ditzen *et al*., 2009; Schneiderman *et al*., 2012). The behavioral and neural effects on response to long-term romantic partners of manipulating individuals’ oxytocin levels have been empirically examined (Scheele *et al*., 2013). In both a discovery and a replication study, either instranasal oxytocin or a placebo was administered to heterosexual male participants in long-term romantic relationships. Oxytocin enhanced the positive behavioral bias towards romantic partner photos (measured by ratings of attractiveness against objectively matched controls of unfamiliar or familiar others). Further, results showed a parallel neural response, as VTA and NAcc were recruited for romantic partners over unfamiliar others in the discovery study. In the replication study, familiar other faces were introduced as a social control; here, oxytocin similarly enhanced the neural response to partners over familiar others in left NAcc and right putamen.

#### Emotion regulation: close others’ capacity to serve as safe havens is manifest in the brain

The regulation of emotion associated with thinking of a close other highlights the role of other neural regions within the limbic system, such as ACC and the insula (Coan *et al*., 2006; Younger *et al*., 2010; Eisenberger *et al*., 2011; Beckes *et al*., 2013). Studies assessing affect regulation often utilized threat paradigms, manipulating participants’ anticipation or experience of pain while in the scanner. The experience of threat can be brought on by a variety of experimental stimuli, such as minor electrical shock, hot or cold sensations, uncomfortable pressure applications or display of anxiety-provoking images or words. Yet, the underlying principle of emotion regulation provided by a close other representation is common to all of these experiments and to adult attachment theory; in the face of stressors, individuals utilize their attachment figure mental representations as safe havens and may show separation distress if this comfort is not available.

Coan, Schaefer and Davidson conducted their seminal `hand-holding‘ study in 2006. They examined spouse *vs* stranger hand-holding when participants were faced with anticipation of a painful experience in the scanner. The authors found patterns of reduced activation in several threat-responsive regions of interest; they observed that spousal hand-holding (in other words, spouse-related attenuation of threat) was associated with decreased activation in right dorsolateral prefrontal cortex, left caudate and NAcc, whereas decreased activation of ventral ACC and posterior cingulate cortex (PCC) was shown for both spouse- and stranger-related attenuation of threat. Using a similar paradigm, modified to tap individuals’ abstract mental representations of non-physically present partners, another experiment had female participants view partner *vs* stranger pictures while receiving painful heat stimulations (Eisenberger *et al*., 2011). Results showed reduced dorsal ACC and anterior insula activation in the partner picture condition. Additionally, results suggested a role for ventromedial prefrontal cortex (vmPFC) engagement in response to partners; increased vmPFC activity when viewing partner photographs was associated with higher perceived support from the partner, longer relationship lengths, reduced subjective ratings of pain and decreased activity in pain-related neural regions such as ACC and insula. Taken together, these findings lend support to a potential neural mechanism underlying the safe haven role of attachment figure mental representations. Attachment figures often serve as safe havens, or responsive people that individuals turn to for comfort in times of distress; the research described above explains the coupling of behavioral responses to attachment figure support with neural reduction of threat and pain.

Several other studies have considered the reversal of roles in affect regulation, investigating how our brains manage perceptions of threat to close others (*vs* to ourselves). Incorporating elements of emotion regulation research and cognitive neuroscientific methods, one study applied a mild electric shock paradigm to look at self-focused threat, *vs* close friend-focused threat or stranger-focused threat (Beckes *et al*., 2013). Significant conjunctions between the threat-to-self and threat-to-friend conditions were observed in anterior insula, putamen and supramarginal gyrus. Studies on the process of giving and receiving emotional support to close relationship partners also exemplify the significant emotion-regulatory capacities of attachment figure representations. A series of studies examining support-giving, support-receiving and even feelings of loneliness further demonstrated the unique response of the ventral striatum to representations of long-term-romantic partners (Inagaki and Eisenberger, 2012; Inagaki *et al*., 2015; Inagaki *et al*., 2016). Taken together, this social neuroscientific research on close other neural representations closely aligns with the tenets of adult attachment theory, moving the field towards an empirically supported neuroscience of attachment.

### MNS: resonance with another’s thoughts and feelings

A smaller group of neuroscientists have asserted the potential role of the MNS in representing close others (Ortigue and Bianchi-Demicheli, 2008; Petrican *et al*., 2015). The MNS—in particular, a collection of neurons within premotor cortex—is thought to play an important role in the ability to understand others’ actions, both in humans and other primates (for review, see van Overwalle and Baetens, 2009 and Ortigue, 2010). One context in which the role of the MNS has been assessed was that of neural responsiveness to a spouse’s incongruent emotions; presumably, our closest relationships might be characterized by sensitivity to when a partner’s feelings may be incongruous with one’s own. One recent study examined older adult female participants in long-term marriages, asking them to make trait judgments about either their spouse’s or a stranger’s affect in the presence of incongruent verbal and non-verbal cues (Petrican *et al*., 2015). Greater activity in putative MNS areas, such as the inferior parietal lobules, was associated only with processing a spouse’s, but not a stranger’s, non-verbal cues when the target’s behavior was positive while in a negative (incongruous) context. Although this line of research shows promise for our growing understanding of the complex role played by the MNS in relating to a close other, researchers have strongly cautioned against over-interpretation of MNS activation (e.g. Caramazza *et al*., 2014), and there are substantial questions remaining about the interaction of this system with others, such as the default network.

### Default network: mentalization for self and others

Cognitive neuroscientists have consistently found activation within a collection of functionally-connected brain regions known as the default network to be associated with social-other mental representations. Core brain areas within the canonical default network include the medial temporal lobes, medial prefrontal cortex (mPFC), PCC, lateral prefrontal cortex, lateral temporal cortices (LTC) and lateral parietal cortices (Buckner and Carroll, 2007; Spreng *et al*., 2009). Default network activity is thought to support many aspects of social cognition. As social beings, we use our own experiences to generate social conceptual knowledge which, in turn, allows us to develop and implement strategic social behaviors reliant on default network function (Spreng and Mar, 2012). For example, the integrity of vmPFC predicts the ability to retrieve impressions of others (Cassidy and Gutchess, 2012), and attributional decisions and judgments of others’ emotional states recruit vmPFC (Haas *et al*., 2015).

The default network also enables us to imagine the experiences of others. In one study, participants were taught the personalities (based on two dimensions of agreeableness and extraversion) of four characters (Hassabis *et al*., 2014). They then imagined those characters’ behaviors across different situations. Results showed that activity in the mPFC reliably predicted which characters the participants were imagining. A number of recent studies have built upon this work, providing substantial evidence for neural mechanisms underlying our ability to understand and predict social others’ mental states (e.g. Thornton and Mitchell, 2017; Thornton *et al*., 2018, 2019). Person-specific patterns of brain activity have even been found for personally familiar others, specifically within right ventral lateral PFC, dorsal PFC, medial precentral gyrus and posterior insula (Thornton and Mitchell, 2017).

Investigations into how the brain represents and navigates social distance have also demonstrated engagement of default network nodes including PCC and LTC. One such study (Tavares *et al*., 2015) presented fictional characters in a virtual role-playing game and collected participants’ self-reported perception of the characters’ power-ranking (i.e. competence, dominance and hierarchy) and affiliation (i.e. warmth, intimacy, trustworthiness and love)—two dimensions of social distance. Results conceptualized these two relational dimensions as vectors in social space, and PCC activity was specifically associated with social distance, or vector length. Another recent study examined the spontaneous encoding of social distance from familiar others within a complex social network (Parkinson *et al*., 2017). This network, comprising a cohort of graduate students, was quantitatively characterized by the authors in several ways, including degrees of social distance (e.g. friend, friend-of-a-friend, etc.). Students in the experiment viewed videos of classmates from this cohort during an fMRI scan. Here the mental representation of social distance recruited posterior LTC as well as lateral posterior superior temporal cortex. These studies provide insight into how we encode distance from others in our social networks, including those with whom we have strong affiliation or familiarity.

Taken together, this body of literature has effectively set the stage for cognitive-neuroscientific investigations of the specific dynamics of unique, close social relationships. In studies specific to close other neural representations, regions of interest within the default network include the mPFC and PCC (Gobbini *et al*., 2004; Heatherton *et al*. 2006; Mitchell *et al*., 2006; Krienen *et al*., 2010; Tacikowski *et al*., 2011, 2013; Wang *et al*., 2012; Laurita *et al*., 2017; Laurita *et al*., 2018). Early work conceptualized social proximity as a function of one of two factors: familiarity or similarity. Participants in the earliest of these studies viewed faces of personally familiar people (relatives and friends), familiar famous individuals (such as public leaders or actors) and strangers (Gobbini *et al*., 2004). Viewing personally familiar faces—contrasted against both famous familiar faces and strangers—was associated with a pattern of neural response in bilateral PCC and precuneus. The authors interpreted their results as evidence for close other `person knowledge‘ in the brain, supporting past findings implicating these regions in processes such as theory of mind for well-known others. MPFC activity also assisted in differentiating similar other representations from dissimilar other representations during a trait-judgement task (Mitchell *et al*., 2006). More specifically, ventral mPFC was recruited here for self-referential and similar other related thought, whereas more dorsal mPFC regions were active for thought regarding dissimilar others. This finding prompted interest in determining how default network activity supports a kind of `simulation‘ of the internal mental states of others and how it might selectivity do so for socially proximate others.

Within the literature on mentalizing (or, imagining the thoughts or feelings of others), a few studies have initiated a focus on the role of mPFC and PCC in differentiating close other from stranger or from self-representations. Some have asserted that the representation of self is `special‘, uniquely recruiting mPFC in contrast to representations of intimately known others (e.g. Heatherton *et al*., 2006), and that representations of one’s own mental states are more distinct than those of both close and less-close others’ mental states (Thornton *et al*., 2018). In 2010, Krienen, Tu and Buckner advanced this earlier work. Participants in their study made judgments about personal preferences in response to facial images of close friends *vs* strangers. They found, for the first time, that friends *vs* strangers yielded a network of brain regions including mPFC, PCC/ retrosplenial cortex, inferior parietal lobe, lateral temporal cortex and medial temporal lobe (Krienen *et al*., 2010).

In the years since this study, further research has provided more nuance to understanding the role of the default network in representing close others. These findings have been extended to collectivistic cultures; thinking of certain close others such as mothers or the self (even over best friends or fathers) in a trait judgment task yielded higher mPFC and ACC activity (Wang *et al*., 2012). A similar pattern of results has also been found using a target name-viewing paradigm (Tacikowski *et al*., 2011) and in extending this paradigm across the modalities of viewing and listening to names (Tacikowki *et al*., 2013).

Considering the rapidly growing evidence for the default network’s role in representing close others, we were interested in how this network, as well as other brain regions and networks, may respond to relationships of differential closeness. More specifically, we wanted to learn how the brain might differentially represent those individuals who serve as our primary attachment figures. To begin answering these questions, we recently explored the neural representation of known others along a continuum of attachment using fMRI (Laurita *et al.*, 2017). In this experiment, heterosexual adults in romantic relationships for over 2 years were asked to make trait judgments for a romantic partner, parent, close friend, familiar acquaintance and self during an fMRI scan. Across all social-other and the self conditions, in contrast to a motor control condition, trait judgments engaged the default network and lateral prefrontal cortex. Judgments about oneself and attached romantic partner additionally recruited anterior and middle cingulate cortex and anterior insula, relative to parent and close friend. These results provided novel evidence that mentalizing about primary attachment figures—here, romantic partners—engages the default and salience networks. Salience network regions such as anterior cingulate and anterior insula detect internal and external events that are personally meaningful and interact with the default network to represent internal events (Uddin *et al*., 2015; Christoff *et al*., 2016). The results of our study showed this interaction by way of the unique patterns of neural response to attached romantic partners and to the self. We concluded that, while the default network is recruited for construction and utilization of social representations, the salience network selectively attunes us to the most meaningful of these representations—those of primary attachment figures.

In a second study, we examined both young and older adults’ (mean age = 67 years old) social representations, utilizing the same trait judgment fMRI paradigm. In particular, we were interested in how individuals neurally represent some of their closest reciprocal relationships—those with parents (young adults) and children (older adults)—and how these representations reflect perceived closeness or attachment to those figures. Patterns of neural activation for mentalizing about a parent or child significantly varied as a function of attachment; interestingly, we found that the more attached one feels to their parent/child, the lower brain activity was observed in brain regions such as ACC, left amygdala hippocampus, anterior and posterior insula, PCC, and the putative occipital face area, suggesting that bringing to mind one’s attachment figure requires less engagement of these brain regions often recruited for distress relief, memory and facial processing.

Our approach across these studies has several innovations for work on known and, specifically, close others. First, our experimental stimuli included the names of real individuals who were highly relevant to each participant. Second, unlike past studies utilizing passive fMRI tasks (such as free-viewing photos of known others), our paradigm required that participants actively mentalize about each of the social targets. Third, we included numerous self-report measures in an attempt to better describe and characterize participants’ relationships with their social targets; one such measure, for example, provided us with information about who participants’ (primary) attachment figures were. We were able to provide initial evidence that the representation of adult attachment is a distinguishing feature of the neural activation differences in social cognition.

### Cortical and subcortical interactions support representation of close others

If one overarching conclusion emerges from fMRI studies of close other representations, it is that we are now arriving at a novel, cross-disciplinary neuroscience of close relationships—yet from several very different perspectives. Just as our mental representations of close others reflect the complexity of rich person-knowledge, emotionally salient memories and unique regulatory capabilities, the neural regions and networks responsible for carrying close other representations are complex, as well. Bringing together past findings, these neural regions and networks appear to include: dopamine-rich regions sensitive to partner reward such as VTA, NAcc and putamen; threat-responsive regions sensitive to partner comfort such as ACC and insula; mirror neuron regions sensitive to a partner’s inner states such as inferior parietal lobules; default network regions sensitive to mentalizing about a partner such as mPFC and PCC; and salience network regions sensitive to meaningful cues associated with a partner such as anterior insula and anterior cingulate.

**Fig. 1:**
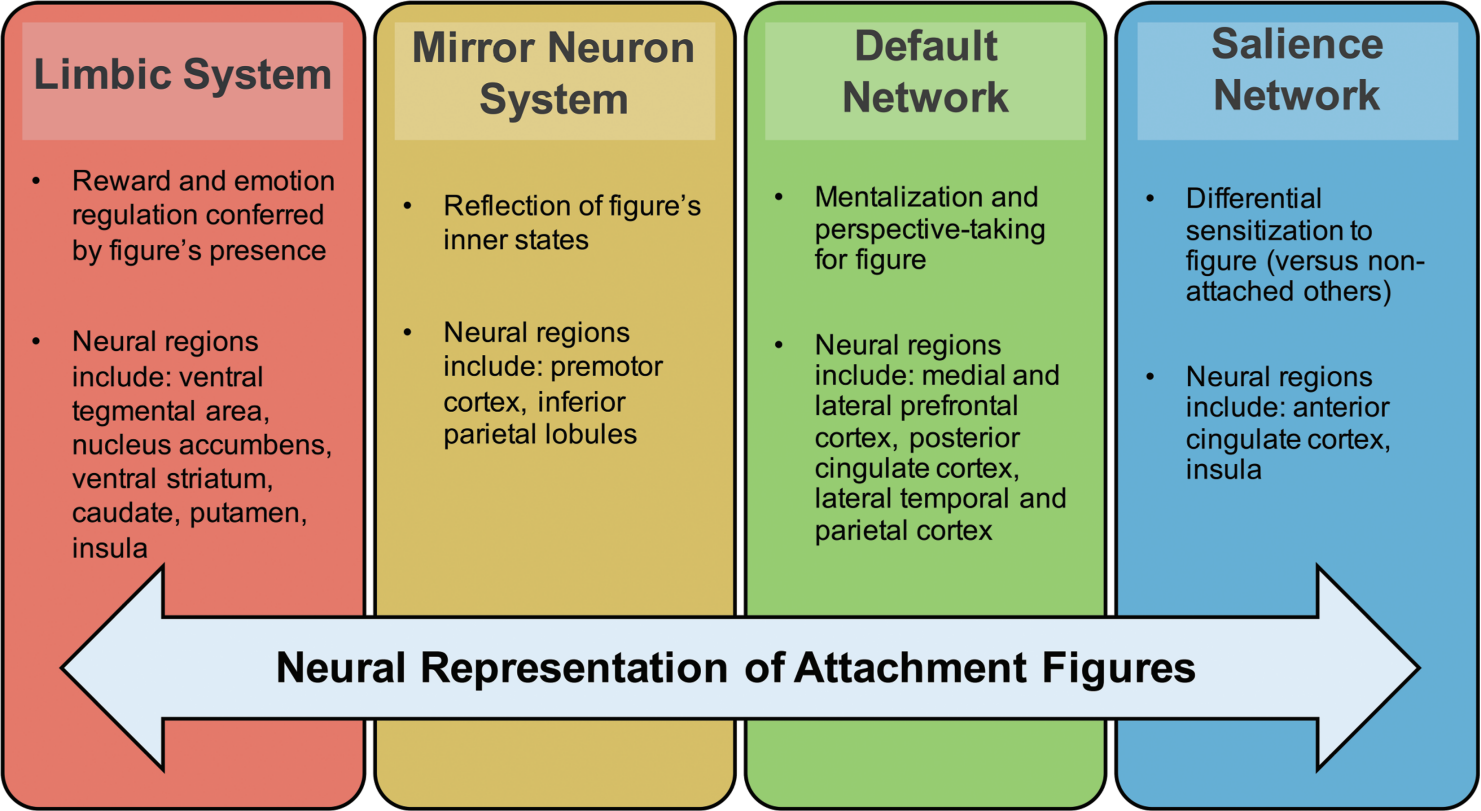
Integrated model of the neural regions and networks recruited in representing attachment figures.

This integrative model (Figure 1) is supported not only by the individual contributions of each of the task-based fMRI studies discussed in this section but also by recent resting state functional connectivity analyses of `in love‘ participants (Song *et al*., 2015). Being deeply `in love‘ may be associated with changes in the functional architecture of the brain, specifically measured by increased functional connectivity within a network of regions important for reward, motivation and emotion regulation (including the dorsal ACC, caudate, NAcc and insula) and, separately, within another network of `social cognition‘ regions resembling the canonical default network (including the PCC, mPFC, precuneus, temporo-parietal junction and inferior parietal lobe; Song *et al*., 2015). Our synthesis shows the interplay between cortical and subcortical regions of the brain, all necessary and each playing different roles to support distinct facets of our complex representations of close others. However, to be able to draw meaningful conclusions from these findings, it is important to refer back to their methodological discrepancies.

## Methodological differences characterize existing research

In the next section of this review, we employ a different approach towards integrating this literature. Here we look across recent fMRI studies of close other mental representations with respect to their methods. Of particular interest to us are experimental parameters reflecting how researchers measure `closeness‘ when studying neural responses to social cognitive stimuli. Here, we discuss the following: usage of terminology relating to close relationships; collection of relationship length data; self-report measures of closeness with relationship partners; social cognitive tasks used in the MRI scanner; and specific condition contrasts used in BOLD fMRI data analysis. We also track sample sizes, as well as gender, sexual orientation and age composition of these studies’ samples.

### Terminology

Even a cursory glance at the terminology used by existing studies provides some rationale for their diverse findings. Authors use varied, but specific, language to operationally define their constructs of interest. They often continue to use this language in reporting and promoting research findings in their articles. Of the studies we surveyed, a majority describe their construct of interest as `romantic love‘ (e.g. Aron *et al*., 2005; Inagaki and Eisenberger, 2012; Acevedo *et al*., 2012; Langeslag *et al*., 2014). Other studies focus on `close others‘ or `close relationships‘ (e.g. Krienen *et al*., 2010), `(personally) familiar others’ (e.g. Beckes *et al*., 2013; Thornton and Mitchell, 2017), `attachment figures‘ (e.g. Eisenberger *et al*., 2011; Laurita *et al.*, 2017), or `significant others‘ (e.g. Tacikowski *et al*., 2011). These discrepancies in terminology preclude meaningful integration of findings across concepts and fields. With various studies employing vastly different—and, at times, ambiguous—terminology, we cannot assume that these studies assess one consistent construct of close other neural representations.

### Relationship length

Relationship length is an essential descriptive statistic in such investigations. A social relationship is comprised of countless salient memories amassed over its course—whether that is a few weeks or numerous years. We assume that close other neural representations would not be identical across differing relationship timepoints. Relationship length is also meaningfully related to the measurement of attachment; while infatuation peaks within the first year of a romantic relationship, behavioral features (e.g. safe haven and secure base) indicating that a full-fledged attachment bond has formed may not be completely present until 2 years or more into the relationship (Hazan and Shaver, 1987). Accurately portraying relationship length plays a critical role in informing the relationship between behavioral shifts over the timecourse of a relationship and neural representation changes.

Yet, the existing literature reveals a lack of reported relationship length data. Half of the studies reviewed do not report any relationship length descriptive statistics for the target close relationships. For those who do report this variable, average (arithmetic mean) length of relationships with target relationship partner range from a few months (Langeslag *et al*., 2014, <9 months; Stoessel *et al*., 2011, <6 months; Younger *et al*., 2010, <9 months) to many years (Acevedo *et al*., 2012, 21 years; Petrican *et al*., 2015, 40.17 years). Several studies list a minimum or maximum relationship length as participation criteria but do not report average relationship lengths. Still more studies do provide descriptive statistics of their samples’ relationship duration. Lastly, the terminology used to describe relationship length often does not capture the nuances inherent to this variable. For example, not all authors distinguished between how long ago participants may have starting dating or married *vs* first met their current relationship partners. Although longitudinal research that compares neural activation patterns at different relationship timepoints is minimal, the existing work does show notable trends in the recruitment of neural regions and networks related to relationship length (Xu *et al*., 2011; Xu *et al*., 2012). Current fMRI research does not recruit or report on a wide-enough range of participant relationship lengths to capture a full understanding of how this variable relates to other neural and behavioral outcome variables.

### Self-report measures of `closeness’

Relationship length data alone, while important to characterize the expected relationship status represented by a sample of participants, is not sufficient to characterize an individual’s feelings of closeness or attachment with his or her partner. It is necessary to include self-report measures to capture participants’ emotions and thoughts regarding their relationships. Collecting these data helps provide an accurate sense of what participants’ social neural representations may indicate. Just under half of the studies we reviewed do not describe any specific self-report measures used to assess relationship closeness or quality. Most who have administered self-report closeness measures rely heavily on the Passionate Love Scale (PLS; Hatfield and Sprecher, 1986). Many studies that include a relationship closeness self-report measure administered only the PLS. The PLS assesses cognitive, emotional and behavioral components of passionate love. The Likert-type items on this scale probe for partner preoccupation, idealization, physical attraction and desire (Hatfield and Sprecher, 1986). The PLS is not an ideal proxy for relationship closeness, as it focuses entirely on participants’ feelings of infatuation or passionate love. In fact, PLS items more closely represent the documented `symptoms‘ of infatuation (Tennov, 1979) than a durable pair bond. Additionally, asking participants to complete only the PLS about their romantic partner could lead to priming neural responses with cues of high reward and approach motivation.

A few studies (e.g. Acevedo *et al*., 2012; Beckes *et al*., 2013; Laurita *et al*., 2017) utilize the Inclusion of Other in the Self (IOS) scale (Aron *et al*., 1992). The IOS is a single-item pictorial measure of closeness and interconnectedness in dyads. The seven instances of two overlapping circles of the IOS range from mutually exclusive to highly overlapping in appearance (Aron *et al*., 1992). The IOS is a direct self-report measure of perceived closeness with relationship partners, as it is a visual representation of how individuals think of their partners and themselves. Yet, there is little space for objective clarification of responses to the IOS. It is possible to view the highly overlapping circles as a negative, enmeshed state not representative of an ideal close relationship.

One study (Acevedo *et al*., 2012), utilizes the PLS, the IOS, the Eros subscale of the Love Attitudes Scale (LAS; Hendrick and Hendrick, 1986), and the Friendship-Based Love Scale (FBLS) (Grote and Frieze, 1994). The FBLS is intended to measure comfortable, affectionate, trusting love for a likable partner, based on a deep sense of friendship. The FBLS is a nine-item Likert-type measure (Grote and Frieze, 1994). While the FBLS is a well-suited complement to the PLS, it does not measure all the components of closeness on its own. The Eros subscale of the LAS assesses levels of passionate love—initial attraction and perceived `chemistry‘ for instance—in one’s relationship with a romantic partner (Hendrick and Hendrick, 1986). In our own recent studies (Laurita *et al*., 2017, 2018), we took a similar approach to Acevedo *et al*. (2012) in collecting self-report data, as we administered a comprehensive relationship battery (see Appendix A).

Many studies implement other measures related to relationship closeness, such as social distance, or to different relationship quality factors such as satisfaction. Several recent studies conceptualize closeness as a quantitatively `short‘ social distance, and utilize metrics of social network connection to characterize participants’ relationships with familiar others on spectra of closeness (e.g. Tavares et al., 2015; Parkinson *et al*., 2017; Thornton and Mitchell, 2017; Thornton *et al*., 2018). Of the studies that do administer other self-report closeness measures, only a few report the resulting behavioral data (e.g. Beckes *et al*., 2013; Tavares *et al*., 2015; Laurita *et al.*, 2017, 2018; Parkinson *et al.*, 2017; Thornton and Mitchell, 2017; Thornton *et al*., 2018) or utilize participants’ responses as variables in their neuroimaging analyses (e.g. Acevedo *et al*., 2012; Tavares *et al*., 2015; Parkinson *et al*., 2017; Thornton and Mitchell, 2017; Thornton *et al*., 2018; Laurita *et al*., 2018). The inconsistencies in usage of self-report closeness measures restrict our ability to make conclusive statements about close other representations in the brain. Collectively, we have not defined what is meant by `close‘ with respect to social relationship representations.

### Social cognitive tasks used in the MRI scanner

There was also great variety seen in the tasks used to evoke neural representations of close others. Most of the studies we examined implement an experimental paradigm in which participants view facial images or videos of their target relationship partner(s) *vs* control images (e.g. Zeki and Romaya, 2010; Acevedo, 2012; Scheele *et al*., 2013; Inagaki *et al*., 2016; Parkinson *et al*., 2017). Within this category, there are several variations on the social cognitive task used, including unpleasant heat stimulations paired with the various facial images (Eisenberger *et al*., 2011), one-back repetition tests (Gobbini *et al*., 2004) and oddball tasks with photos as targets or distractors (Langeslag *et al*., 2014). Beyond facial image viewing, other tasks include trait judgment of partner *vs* others (Heatherton *et al*., 2006; Krienen *et al*., 2010; Wang *et al*., 2012; Tavares *et al*., 2015; Laurita *et al*., 2017), consideration of others’ mental states (e.g. Thornton *et al*., 2018), and support giving or receiving from partner *vs* others (Coan *et al*., 2006; Inagaki and Eisenberger, 2011). One study used the administration of oxytocin to participants as an independent variable in their experiment (Scheele *et al*., 2013). Another recent study does not include a specific social cognitive task but instead looks at how romantic love may be associated with neural functional architecture, by assessing functional connectivity in a resting state scan for `in love‘ participants (Song *et al*., 2015).

It is probable that the diverse social cognitive tasks we choose lead to distinct patterns of activation in the brain. For instance, we would expect to see different findings in response to a partner trait-judgement task (e.g. Krienen *et al*., 2010) *vs* a partner hand-holding experimental paradigm (Beckes *et al*., 2013). Although these tasks may be assessing the same construct of cognitive representations of close relationships, the relevant representations are likely activated for distinct motivational purposes across the studies mentioned above.

### Specific condition contrasts used in BOLD fMRI data analysis

Each of the studies in the above section includes control conditions for activation contrasts within their BOLD fMRI data. Most studies use exclusively social contrast conditions such as a less-close friend, a highly familiar other, an acquaintance, a known famous figure or a complete stranger. Others include non-social controls—such as the categorization of a typographical font (Wang *et al*., 2012). When studying social closeness, it is crucial to control for as many other interpersonal factors as possible. For instance, including conditions for a familiar but non-close other or a friend known for an equal amount of years as a romantic partner would allow for better isolation of the social closeness variable.

Scheele *et al*. (2013) demonstrate the importance of including a variety of social contrasts, introducing famous-other and then familiar-other faces as specific controls to romantic partners faces in their discovery and replication studies, respectively. This methodological choice allowed them to interpret their activational results as specific to close others—not simply familiar others. In our aforementioned study (Laurita *et al*., 2017), we also examine close other representations using several relevant social condition contrasts. By including a variety of social contrast conditions in our study, we were able to isolate patterns of neural activity specific to primary attachment figure representations. However, it is increasingly clear that few neuroimaging studies have systematically assessed the continuum of personal relatedness and attachment in this way.

### Size and demographic composition of samples

Samples range from 10 (Gobbini *et al*., 2004) to 98 (Krienen *et al*., 2010) subjects scanned. Several studies only scanned heterosexual females as part of partner-pairs (e.g. Coan *et al*., 2006; Petrican *et al*., 2015), and one study included only heterosexual males in romantic relationships (Scheele *et al*., 2013). Most studies include primarily college-aged, young adults, with only a few examining older adults (Acevedo *et al*., 2012; Petrican *et al*., 2015). These inconsistencies and shortcomings in study demographics further cloud our understanding of neural representations of close others. It is clear that we need to direct attention and resources towards studying men, non-exclusively-heterosexual individuals and older adults. The close relationships literature is rife with gender differences, and there is substantial reason to believe that neural representations of close others may be different across genders (e.g. Hendrick and Hendrick, 1995; Burleson, 2003; Diamond, 2003). Most of the existing literature on sex differences focuses on differences in attachment styles. For example, Del Guidice conducted a meta-analysis in 2011, finding that males show higher avoidance and lower anxiety in attachment than do females. Zeki and Romaya (2010) found no gender or sexual orientation differences in brain activation. Yet, undiscovered differences could certainly exist.

## Recommendations, and a proposal for a standardized assessment battery

Although we are far from achieving the goal of cohesive integration of cognitive and social theories of relationships, we can progress toward consistent utilization of theoretically and empirically based methodological procedures and increased awareness of attachment theory’s applications. Here, we offer a series of recommendations (see Table 1) designed to promote an interdisciplinary approach for mapping the neural representation of our closest relationships, conceptualized as attachment bonds:

**Table 1:** Recommendations to promote an interdisciplinary approach to close relationship neuroscience

Our recommendation	One example of implementation
1. Increased awareness of attachment theory and social neuroscience as reciprocally guiding frameworks through cross-disciplinary collaborations	Initiate research partnership between social psychologist and cognitive neuroscientist
2. More focused participant recruitment to capture the full spectrum of social relationships	Recruit participants who, based on self-report data, maintain attachment relationships with a romantic partner and/or a parent
3. Design of neuroimaging tasks that directly capture how participants behaviorally utilize attachment figure mental representations	Utilize a social-cognitive task that requires active mentalization, such as trait judgment
4. Necessary inclusion of social controls in neuroimaging tasks	Include targets of romantic partners, family members, close friends, acquaintances, famous figures, strangers and self as control conditions
5. Implementation of rigorous methodological practices needed for statistical power in neuroimaging studies, including larger sample size and proper reporting of brain and behavioral data	Collect and report descriptive statistics for all self-report data pertaining to relationships
6. Utilization of a standardized battery of self-report measures	Include measures found in Appendix A

### (i) Increased awareness of attachment theory and social neuroscience as reciprocally guiding frameworks through cross-disciplinary collaborations

In this review, we discuss the applicability of adult attachment theory to the study of neural representations of close others. Since attachment bonds in adulthood are quantifiable and their associated behaviors, emotions and cognitions are already well studied, we believe this to be a fruitful approach to categorizing our closest social relationships. Only once relationships are adequately described and categorized can we expect to find reliable patterns of neural activity that reliably underlie their representations. We also raise the inextricable notion that our understanding of social neuroscience can in turn guide investigations of attachment figure mental representations, a smaller subset of the many social-other neural representations individuals form and maintain.

Until recent years, there has been only minimal evidence of cross-talk between social-psychological theorists and social-cognitive neuroscientists regarding the study of close relationship representations. Despite significant overlap in researchers’ topics and populations of interest, few examples of collaborative projects exist, to date (e.g. Eisenberger *et al*., 2011; Acevedo *et al*., 2012; Laurita *et al*., 2017). As part of this recommendation, we hope to promote cross-disciplinary collaborations that bring together experts from these fields. We believe that such partnerships would enable the effective application of attachment theory within social neuroscience and would yield clearer neuroimaging results.

### (ii) More focused participant recruitment to capture the full spectrum of social relationships

In human subjects research, it is often difficult to fully control for pre-existing characteristics that may be related to study outcomes. Yet, it is necessary that we do what we can to improve construct validity and reliability. We should recognize the connection between accurate self-report of data describing participants’ close relationships and how we eventually characterize a representative sample. One way to move towards greater consistency within a sample and generalizability to other samples would be to include relationship criteria as part of more focused recruitment strategies. For example, researchers could recruit participants who maintain attachment relationships with a romantic partner and/or a parent (according to self-report and/or relationship length data).

### (iii) Design of neuroimaging tasks that directly capture how participants behaviorally utilize attachment figure mental representations

A study’s motivations are inherently connected to its results through the careful design of its experimental paradigm. As evidenced by the variety of fMRI tasks we cover in the present review, there is space for both replications and constructions of different tasks in future research. We argue that the design of neuroimaging tasks can be better-informed by understanding the affective, behavioral and cognitive processes involved in representing close others. We recommend that researchers explore different types of tasks that require active mentalization about or utilization of attachment figure representations. Passive tasks will not require participants to draw upon their attachment figure representations in replicable or consistent ways. Examples of active mentalization tasks include, but are not limited to, trait judgement, or social autobiographical memory, accompanied by target name or photograph prompts that are individualized for each participant. Other rigorous designs that would require utilization of attachment figure representations include real or simulated presence of attachment figures in threat-induction paradigms (e.g. hand-holding task).

### (iv) Necessary inclusion of social controls in neuroimaging tasks

We believe this point is important enough to be a separate recommendation; it is challenging to draw conclusions about any close other neural representations if social contrasts are not intentionally included. Implications of task-based fMRI findings rely on our ability to compare patterns of activation across different conditions. We recommend that all studies of close other representations include targets such as romantic partners, family members, close friends, acquaintances, famous figures, strangers and the self as control conditions that are social in nature and possess ecological validity. Within the broad social category of known others, there may be substantial differences along the dimensions of closeness and familiarity. To parse out behavioral and neural differences between several known others, studies could require participants to think of specific exemplars of each of the following dimensions: an attached romantic partner or family member, a friend with whom the participant is close and familiar but not attached, a familiar but not close acquaintance and a known but not close or familiar famous figure. Ideally, future fMRI studies will capture patterns of brain activity for more complete spectra of familiarity and closeness.

### (v) Implementation of rigorous methodological practices needed for statistical power in neuroimaging studies, including larger sample size and proper reporting of brain and behavioral data

Increased interest in neural correlates of social psychological constructs must go hand-in-hand with adherence to rigorous methodological practices needed for neuroimaging studies (for review, see Yarkoni, 2009; Button *et al*., 2013; Mar *et al*., 2013; Poldrack *et al*., 2017). It is not reasonable to interpret individual differences in a sample of 30 fMRI participants or to draw any conclusions from a sample of 10; such investigations can actively muddle this emerging field of close relationship neuroscience. Likewise, minimal inclusion of behavioral data reflects an inadequate understanding of the overlap between psychology and neuroscience in understanding close relationships. Both brain data and behavioral data should be collected and reported in accordance with the highest standards of both disciplines.

### (vi) Utilization of a standardized battery of self-report measures

Importantly, we need to administer self-report measures that answer numerous questions about participants’ cognitions, behaviors and emotions within the context of their relationships. Some of the questions we would certainly want future participants to answer include: To whom are participants attached? How do participants view their social closeness with specific others? Toward whom do participants feel passionate love? Toward whom do participants feel companionate love? What attachment style do participants show in their current romantic relationships? How committed, satisfied and invested do participants feel in their current romantic relationships? How long have participants been in relationships with their romantic partners? How long have participants known specific other people?

Answers to these questions could let us know, on a basic level, the core drivers of regional and network brain activation differences. If we implement a standard battery of measures addressing these and other questions across studies, we can begin to tap into a unified cognitive construct of attachment figure mental representations. Such a standard battery can provide a clear picture of the content of attachment figure mental representations, the attachment `status‘ and style, and the specific ways in which attachment figure representations are different from other social representations.

Our proposed standard battery can be found in Appendix A. This compilation of close relationship self-report measures includes the WHOTO (Hazan *et al*., 1991; Fraley and Davis, 1997), IOS (Aron *et al*., 1992), PLS (Hatfield and Sprecher, 1986), FBLS (Grote and Frieze, 1994), Partner-Specific Experiences in Close Relationships Scale (ECR-R-PS; Fraley *et al*., 2000) and brief partner-specific and general relationship questionnaires of our own design. Each measure serves a specific purpose in providing clear, attachment-related information about an adult’s close relationships. The WHOTO (Hazan *et al*., 1991; Fraley and Davis, 1997) is an attachment functions measure that determines the people with whom subjects display attachment relationships. The items are based on the four attachment-related components or features: proximity seeking, separation distress, safe haven and secure base. Subjects list up to four most important figures in their lives by generic labels (e.g. `mother‘, `husband‘) for each of the 10 items. The ECR-R-PS (Fraley *et al*., 2000), is a measure designed to assess individual differences with respect to attachment-related anxiety and avoidance. This partner-specific version assesses these differences within the context of subjects’ current romantic relationships. Responses to 10 items are on a seven-point Likert scale.

Lastly, we include brief questionnaires in order to gain consistent self-report data about variables such as relationship length. The partner specific items assess commitment, exclusivity and satisfaction, whereas the general questions gauge depth of personal knowledge and emotional investment in any kind of relationship. By including each of these measures, in addition to the highly relevant IOS (Aron *et al*., 1992) and complementary PLS (Hatfield and Sprecher, 1986) and FBLS (Grote and Frieze, 1994), we account for whom participants feel the closest to and what their attachment status is. Completion of this battery provides extensive information about participants’ potential attachments to romantic partners, in particular.

## Concluding remarks

Various regions of the brain, including those important for reward, emotional regulation, memory and understanding of others’ actions, are recruited in the activation of mental representations of attachment figures. The neural systems involved in the formation and function of mental representations in adult attachment relationships are understandably complex. In the present work, we call attention to an emerging field of close relationship neuroscience and a gap in its literature that would benefit greatly from increasing collaborations across disciplines.

We have asserted that the developing social neuroscience of attachment is based in both a rich theoretical framework and an increasingly robust collection of empirical studies. Attachment theory suggests that behavior towards certain close others differs fundamentally from behavior towards more distant others. One important characteristic of attachment bonds is the feeling of security associated with attachment figures’ proximity. As individuals undergo conditioning processes over the course of relationship development, the accessibility of attachment figure mental representations supplants the need for physical proximity. Social cognitive neuroimaging studies implicate a wide array of brain systems in supporting close other representations, including those of attachment figures. Engagement of reward systems, as well as the distress-alleviation mechanism within the limbic system, have been implicated in attachment formation and maintenance. Past work also highlights the role of the limbic system in emotional regulation provided by a close other. Memory systems support the encoding and retrieval of person-specific knowledge and social memories necessary to form rich cognitive representations. The default network has also been implicated in differentiating mental representations of oneself and of known others. Lastly, the salience network demonstrates a critical ability to distinguish primary attachment figure representations from other social representations. Past studies have, however, applied diverse approaches to operationalize and measure the constructs of close relationship representations or attachment figure representations.

In light of the excitement and confusion surrounding this new area of research, we have offered a series of recommendations designed to promote an interdisciplinary approach for mapping the neural basis of attachment figure representations. We assert that administration of standardized assessments, including measures of attachment status, style and relationship quality, is necessary to develop comprehensive, reliable and replicable markers of real-world attachment representations. In addition to uniting the contributing neuroimaging fields, future research could include implementation of longitudinal designs investigating which neural structures are sensitive to the affective, behavioral, cognitive and physiological processes involved in attachment figure mental representations. In the future, it could be possible to learn how neural representations of primary attachment figures within a romantic couple or a parent/ child relationship change over the course of a lifetime spent together. As attachment theory includes such a robust developmental component, this type of longitudinal study—or cross-sectional examinations of adolescents or children—could be very fruitful in further deciphering the creation and maintenance of neural representations of attachment figures. We might also be able to examine what kinds of interpersonal experiences, specifically, recruit neural regions of interest within the context of close other mental representations. We already have evidence for how crucial attachment bonds are for our psychological and physical health throughout the life course; with cross-disciplinary communication and sharing of methodological tools, the possibilities to learn more about the brain’s function in these powerful relationships is limitless.

## References

[ref1] Acevedo B.P., Aron A., Fisher H.E., Brown L.L. (2012). Neural correlates of long-term intense romantic love. Social Cognitive and Affective Neuroscience, 7, 145–159. 21208991 10.1093/scan/nsq092 PMC3277362

[ref3] Ainsworth M.D.S., Blehar M.C., Waters E., Wall S. (1978). *Patterns of Attachment: A Psychological Study of the Strange Situation*, Hillsdale, NJ: Erlbaum.

[ref4] Andersen S.M., Cole S.W. (1990). `Do I know you?’: the role of significant others in general social perception. Journal of Personality and Social Psychology, 59, 384–399. doi:10.1037/0022-3514.59.3.384 2231277

[ref5] Aron A., Aron E.N. (1986). Love and the expansion of self: Understanding attraction and satisfaction, Hemisphere Publishing Corp/Harper & Row Publishers.

[ref6] Aron A., Aron E.N., Smollan D. (1992). Inclusion of other in the self scale and the structure of interpersonal closeness. Journal of Personality and Social Psychology, 63, 596–612.

[ref7] Aron A., Fisher H., Mashek D.J., Strong G., Li H., Brown L.L. (2005). Reward, motivation, and emotion systems associated with early-stage intense romantic love. Journal of Neurophysiology, 94, 327–337. 15928068 10.1152/jn.00838.2004

[ref8] Baldwin M.W., Keelan J.P.R., Fehr B., Enns V., Koh-Rangarajoo E. (1996). Social-cognitive conceptualization of attachment working models: availability and accessibility effects. Journal of Personality and Social Psychology, 71, 94.

[ref9] Bartels A., Zeki S. (2000). The neural basis of romantic love. Neuroreport, 11, 3829–3834. 11117499 10.1097/00001756-200011270-00046

[ref11] Beck L.A., Pietromonaco P.R., DeBuse C.J., Powers S.I., Sayer A.G. (2013). Spouses’ attachment pairings predict neuroendocrine, behavioral, and psychological responses to marital conflict. Journal of Personality and Social Psychology, 105, 388. 23773048 10.1037/a0033056 PMC4076153

[ref12] Beckes L., Coan J.A., Hasselmo K. (2013). Familiarity promotes the blurring of self and other in the neural representation of threat. Social Cognitive and Affective Neuroscience, 8, 670–677. 22563005 10.1093/scan/nss046 PMC3739912

[ref13] Bowlby J. (1973). Attachment and loss: Separation, vol. 2 London: The Hogarth Press and the Institute of Psycho-Analysis.

[ref14] Buckner R.L., Carroll D.C. (2007). Self-projection and the brain. Trends in Cognitive Sciences, 11, 49–57. doi: 10.1016/j.tics.2006.11.004 17188554

[ref15] Burleson B.R. (2003). The experience and effects of emotional support: what the study of cultural and gender differences can tell us about close relationships, emotion, and interpersonal communication. Personal Relationships, 10, 1–23.

[ref16] Buss D.M., Schmitt D.P. (1993). Sexual strategies theory: an evolutionary perspective on human mating. Psychological Review, 100, 204. 8483982 10.1037/0033-295x.100.2.204

[ref17] Button K.S., Ioannidis J.P., Mokrysz C., et al. (2013). Power failure: why small sample size undermines the reliability of neuroscience. Nature Reviews Neuroscience, 14, 365–376. 23571845 10.1038/nrn3475

[ref18] Caramazza A., Anzellotti S., Strnad L., Lingnau A. (2014). Embodied cognition and mirror neurons: a critical assessment. Annual Review of Neuroscience, 37, 1–15. 10.1146/annurev-neuro-071013-013950 25032490

[ref19] Cassidy B.S., Gutchess A.H. (2012). Structural variation within the amygdala and ventromedial prefrontal cortex predicts memory for impressions in older adults. Frontiers in Psychology, 3, 319. doi:10.3389/fpsyg.2012.00319 22973250 PMC3428811

[ref20] Christoff K., Irving Z.C., Fox K.C.R., Spreng R.N., Andrews-Hanna J.R. (2016). Mind-wandering as spontaneous thought: a dynamic framework. Nature Reviews Neuroscience, 17, 718–731. 27654862 10.1038/nrn.2016.113

[ref21] Coan J.A., Sbarra D.A. (2015). Social baseline theory: the social regulation of risk and effort. Current Opinion in Psychology, 1, 87–91. 25825706 10.1016/j.copsyc.2014.12.021 PMC4375548

[ref22] Coan J.A., Schaefer H.S., Davidson R.J. (2006). Lending a hand: social regulation of the neural response to threat. Psychological Science, 17, 1032–1039. doi 10.1111/1467-9280.2006.01832.x 17201784

[ref23] Cohen S. (2004). Social relationships and health. American Psychologist, 59, 676. 15554821 10.1037/0003-066X.59.8.676

[ref24] Collins N.L., Feeney B.C. (2004). Working models of attachment shape perceptions of social support: evidence from experimental and observational studies. Journal of Personality and Social Psychology, 87, 363–383. 15382986 10.1037/0022-3514.87.3.363

[ref25] Cornwell E.Y., Waite L.J. (2009). Social disconnectedness, perceived isolation, and health among older adults. Journal of Health and Social Behavior, 50, 31–48. 19413133 10.1177/002214650905000103 PMC2756979

[ref26] Diamond L.M. (2003). What does sexual orientation orient? A biobehavioral model distinguishing romantic love and sexual desire. Psychological Review, 110, 173. 12529061 10.1037/0033-295x.110.1.173

[ref27] Ditzen B., Schaer M., Gabriel B., Bodenmann G., Ehlert U., Heinrichs M. (2009). Intranasal oxytocin increases positive communication and reduces cortisol levels during couple conflict. Biological Psychiatry, 65, 728–731. 19027101 10.1016/j.biopsych.2008.10.011

[ref28] Dunbar R. (1998). The social brain hypothesis. Evolutionary Anthropology, 6, 178–190.

[ref29] Eisenberger N.I., Master S.L., Inagaki T.K. et al (2011). Attachment figures activate a safety signal-related neural region and reduce pain experience. Proceedings of the National Academy of Sciences USA, 108, 11721–11726. doi:10.1073/pnas.1108239108 PMC3136329 21709271

[ref30] Feldman R. (2017). The neurobiology of human attachments. Trends in Cognitive Sciences, 21, 80–99. 28041836 10.1016/j.tics.2016.11.007

[ref31] Fitzsimons G.M., Bargh J.A. (2003). Thinking of you: nonconscious pursuit of interpersonal goals associated with relationship partners. Journal of Personality and Social Psychology, 84, 148. 12518976 PMC3011819

[ref32] Fisher H., Aron A., Brown L.L. (2005). Romantic love: an fMRI study of a neural mechanism for mate choice. Journal of Comparative Neurology, 493, 58–62. 16255001 10.1002/cne.20772

[ref33] Fletcher G.J., Simpson J.A., Campbell L., Overall N.C. (2015). Pair-bonding, romantic love, and evolution the curious case of homo sapiens. Perspectives on Psychological Science, 10, 20–36. 25910380 10.1177/1745691614561683

[ref34] Fraley R.C., Davis K.E. (1997). Attachment formation and transfer in young adults' close friendships and romantic relationships. Personal Relationships, 4, 131–144.

[ref35] Fraley R.C., Waller N.G., Brennan K.A. (2000). An item-response theory analysis of self-report measures of adult attachment. Journal of Personality and Social Psychology, 78, 350–365. 10707340 10.1037//0022-3514.78.2.350

[ref36] Gobbini M.I., Leibenluft E., Santiago N., Haxby J.V. (2004). Social and emotional attachment in the neural representation of faces. Neuroimage, 22, 1628–1635. 15275919 10.1016/j.neuroimage.2004.03.049

[ref37] Grewen K.M., Anderson B.J., Girdler S.S., Light K.C. (2003). Warm partner contact is related to lower cardiovascular reactivity. Behavioral Medicine, 29, 123–130. doi:10.1080/08964280309596065 15206831

[ref38] Grewen K.M., Girdler S.S., Amico J., Light K.C. (2005). Effects of partner support on resting oxytocin, cortisol, norepinephrine, and blood pressure before and after warm partner contact. Psychosomatic Medicine, 67, 531–538. 16046364 10.1097/01.psy.0000170341.88395.47

[ref39] Grote N., Frieze I.H. (1994). The measurement of friendship-based love in intimate relationships. Personal Relationships, 1, 275–300.

[ref40] Günaydin G., Zayas V., Selcuk E., Hazan C. (2012). I like you but I don't know why: objective facial resemblance to significant others influences snap judgments. Journal of Experimental Social Psychology, 48, 350–353.

[ref41] Haas B.W., Anderson I.W., Filkowski M.M. (2015). Interpersonal reactivity and the attribution of emotional reactions. Emotion, 15, 390. 25706827 10.1037/emo0000053

[ref2] Hassabis D., Spreng R.N., Rusu A.A., Robbins C.A., Mar R.A., Schacter D.L. (2014). Imagine all the people: how the brain creates and uses personality models to predict behavior. Cerebral Cortex, 24, 1979–1987. 23463340 10.1093/cercor/bht042 PMC4089378

[ref43] Hatfield E., Sprecher S. (1986) Measuring passionate love in intimate relations). Journal of Adolescence, 9, 383–4l0. 3805440 10.1016/s0140-1971(86)80043-4

[ref44] Hazan C., Shaver P.R. (1987). Romantic love conceptualized as an attachment process. Journal of Personality and Social Psychology, 52, 511–524. 3572722 10.1037//0022-3514.52.3.511

[ref45] Hazan C., Gur-Yaish N., Campa M. (2004). What does it mean to be attached? In: Rholes W.S., Simpson J.A., editors. *Adult Attachment: New Directions and Emerging Issues*, New York: Guilford Press, 55–85.

[ref46] Hazan C., Hutt M.J., Sturgeon J., Bricker T. (1991). The process of relinquishing parents as attachment figures. Paper presented at the biennial meetings of the Society for Research in Child Development, WA: Seattle.

[ref47] Hazan C., Zeifman D. (1999). Pair bonds as attachments. Handbook of Attachment: Theory, Research, and Clinical Applications, New York, NY, US: The Guilford Press, 336–354.

[ref48] Heatherton T.F., Wyland C.L., Macrae C.N., Demos K.E., Denny B.T., Kelley W.M. (2006). Medial prefrontal activity differentiates self from close others. Social Cognitive and Affective Neuroscience, 1, 18–25. 18985097 10.1093/scan/nsl001 PMC2555408

[ref10] Hendrick S.S., Hendrick C. (1986). A theory and method of love. Journal of Personality and Social Psychology, 50, 392.

[ref49] Hendrick S.S., Hendrick C. (1995). Gender differences and similarities in sex and love. Personal Relationships, 2, 55–65.

[ref51] House J.S., Landis K.R., Umberson D. (1988). Social relationships and health. Science, 241, 540–545. doi:10.1126/science.3399889 3399889

[ref52] Inagaki T.K., Eisenberger N.I. (2012). Neural correlates of giving support to a loved one. Psychosomatic Medicine, 74, 3–7. 22071630 10.1097/PSY.0b013e3182359335

[ref53] Inagaki T.K., Haltom K.E.B., Suzuki S., et al. (2016). The neurobiology of giving versus receiving support: the role of stress-related and social reward-related neural activity. Psychosomatic Medicine, 78, 443–453. 26867078 10.1097/PSY.0000000000000302 PMC4851591

[ref54] Inagaki T.K., Muscatell K.A., Moieni M., et al. (2015). Yearning for connection? Loneliness is associated with increased ventral striatum activity to close others. Social Cognitive and Affective Neuroscience, nsv076. 10.1093/scan/nsv076 PMC4927031 26084531

[ref55] Kiecolt-Glaser J.K., Newton T.L. (2001). Marriage and health: his and hers. Psychological Bulletin, 127, 472. 11439708 10.1037/0033-2909.127.4.472

[ref57] Krakauer J.W., Ghazanfar A.A., Gomez-Marin A., MacIver M.A., Poeppel D. (2017). Neuroscience needs behavior: correcting a reductionist bias. Neuron, 93, 480–490. 28182904 10.1016/j.neuron.2016.12.041

[ref58] Krienen F.M., Tu P.C., Buckner R.L. (2010). Clan mentality: evidence that the medial prefrontal cortex responds to close others. The Journal of Neuroscience: The Official Journal of the Society for Neuroscience, 30, 13906–13915. 20943931 10.1523/JNEUROSCI.2180-10.2010 PMC2989424

[ref59] Langeslag S.J., Veen F.M., Röder C.H. (2014). Attention modulates the dorsal striatum response to love stimuli. Human Brain Mapping, 35, 503–512. 23097247 10.1002/hbm.22197 PMC6869091

[ref60] Laurita A.C., Hazan C., Spreng R.N. (2018). Neural signatures of chronic accessibility in parent–adult child attachment bonds. Social Neuroscience, 1–8. 10.1080/17470919.2018.1494037 29949456

[ref61] Laurita A.C., Hazan C., Spreng R.N. (2017). Dissociable patterns of brain activity for mentalizing about known others: a role for attachment. Social Cognitive and Affective Neuroscience, 12, 1072–1082. 28407150 10.1093/scan/nsx040 PMC5490684

[ref62] Love T.M., Enoch M.A., Hodgkinson C.A. et al. (2012). Oxytocin gene polymorphisms influence human dopaminergic function in a sex-dependent manner. Biological Psychiatry, 72, 198–206. 22418012 10.1016/j.biopsych.2012.01.033 PMC3392442

[ref64] Mar R.A., Spreng R.N., DeYoung C.G. (2013). How to produce personality neuroscience research with high statistical power and low additional cost. Cognitive, Affective, & Behavioral Neuroscience, 13, 674–685. 10.3758/s13415-013-0202-6 23982973

[ref65] Mikulincer M., Shaver P.R. (2007). *Attachment in Adulthood: Structure, Dynamics, and Change*, New York, NY: Guilford Press.

[ref67] Mitchell J.P., Macrae C.N., Banaji M.R. (2006). Dissociable medial prefrontal contributions to judgments of similar and dissimilar others. Neuron, 50, 655–663. 16701214 10.1016/j.neuron.2006.03.040

[ref68] Nickerson A.B., Nagle R.J. (2005). Parent and peer attachment in late childhood and early adolescence. The Journal of Early Adolescence, 25, 223–249.

[ref69] Ortigue S., Bianchi-Demicheli F., Patel N., Frum C., Lewis J.W. (2010). Neuroimaging of love: fMRI meta-analysis evidence toward new perspectives in sexual medicine. Journal of Sexual Medicine, 7, 3541–3552 20807326 10.1111/j.1743-6109.2010.01999.x

[ref70] Ortigue S., Bianchi-Demicheli F. (2008). Why is your spouse so predictable? Connecting mirror neuron system and self-expansion model of love. Medical Hypotheses, 71, 941–944. 18722062 10.1016/j.mehy.2008.07.016

[ref71] Parkinson C., Kleinbaum A.M., Wheatley T. (2017). Spontaneous neural encoding of social network position. Nature Human Behaviour, 1, 0072.

[ref72] Petrican R., Rosenbaum R.S., Grady C. (2015). Neural activity patterns evoked by a spouse's incongruent emotional reactions when recalling marriage-relevant experiences. Human Brain Mapping, 36, 4164–4183. 26219536 10.1002/hbm.22909 PMC4898962

[ref73] Pietromonaco P.R., DeBuse C.J., Powers S.I. (2013). Does attachment get under the skin? Adult romantic attachment and cortisol responses to stress. Current Directions in Psychological Science, 22, 63–68. 25309053 10.1177/0963721412463229 PMC4192659

[ref74] Pietromonaco P.R., Feldman Barrett L., Powers S. (2006). Adult attachment theory and affective reactivity and regulation In Snyder D.K., Simpson J.A., Hughes J.N., editors. Emotion Regulation in Families: Pathways to Dysfunction and Health, pp. 57–74. Washington, DC: American Psychological Association. doi:10.1037/11468-003

[ref76] Poldrack R.A., Baker C.I., Durnez J., et al. (2017). Scanning the horizon: towards transparent and reproducible neuroimaging research. Nature Reviews Neuroscience, 18, 115. 28053326 10.1038/nrn.2016.167 PMC6910649

[ref77] Sbarra D.A., Coan J.A. (2018). Relationships and health: the critical role of affective science. Emotion Review, 10(1), 40–54.

[ref78] Scheele D., Wille A., Kendrick K.M., et al. (2013). Oxytocin enhances brain reward system responses in men viewing the face of their female partner. Proceedings of the National Academy of Sciences, 110, 20308–20313. 10.1073/pnas.1314190110 PMC3864312 24277856

[ref80] Schneiderman I., Zagoory-Sharon O., Leckman J.F., Feldman R. (2012). Oxytocin during the initial stages of romantic attachment: relations to couples’ interactive reciprocity. Psychoneuroendocrinology, 37, 1277–1285. 22281209 10.1016/j.psyneuen.2011.12.021 PMC3936960

[ref81] Selcuk E., Zayas V., Günaydin G., Hazan C., Kross E. (2012). Mental representations of attachment figures facilitate recovery following upsetting autobiographical memory recall. Journal of Personality and Social Psychology. Advance online publication, 103, 362. doi: 10.1037/a0028125 22486677

[ref82] Song H., Zou Z., Kou J., et al. (2015). Love-related changes in the brain: a resting-state functional magnetic resonance imaging study. Frontiers in Human Neuroscience, 9, 71. 25762915 10.3389/fnhum.2015.00071 PMC4327739

[ref83] Spreng R.N., Mar R.A. (2012). I remember you: a role for memory in social cognition and the functional neuroanatomy of their interaction. Brain Research, 1428, 43–50. doi: 10.1016/j.brainres.2010.12.024 21172325 PMC3085056

[ref84] Spreng R.N., Mar R.A., and Kim A.S. (2009). The common neural basis of autobiographical memory, prospection, navigation, theory of mind, and the default mode: a quantitative meta-analysis. Journal of Cognitive Neuroscience, 21, 489–510. doi: 10.1162/jocn.2008.21029 18510452

[ref85] Sroufe L.A., Waters E. (1977). Attachment as an organizational construct. Child Development, 48, 1184–1199. doi:10.2307/1128475

[ref86] Stoessel C., Stiller J., Bleich S. et al (2011). Differences and similarities on neuronal activities of people being happily and unhappily in love: a functional magnetic resonance imaging study. Neuropsychobiology, 64, 52–60. 10.1159/000325076[doi] 21606659

[ref87] Tacikowski P., Brechmann A., Marchewka A., Jednorog K., Dobrowolny M., Nowicka A. (2011). Is it about the self or the significance? An fMRI study of self-name recognition. Social Neuroscience, 6, 98–107. 10.1080/17470919.2010.490665[doi] 20602286

[ref88] Tacikowski P., Brechmann A., Nowicka A. (2013). Cross-modal pattern of brain activations associated with the processing of self- and significant other's name. Human Brain Mapping, 34, 2069–2077. 22431327 10.1002/hbm.22048 PMC6869889

[ref89] Tavares R.M., Mendelsohn A., Grossman Y., et al. (2015). A map for social navigation in the human brain. Neuron, 87(1), 231–243. 26139376 10.1016/j.neuron.2015.06.011 PMC4662863

[ref90] Thornton M.A., Mitchell J.P. (2017). Consistent neural activity patterns represent personally familiar people. Journal of Cognitive Neuroscience, 29, 1583–1594. 28557690 10.1162/jocn_a_01151

[ref91] Thornton M.A., Weaverdyck M.E., Mildner J., Tamir D. (2018). People represent their own mental states more distinctly than others’. PsyArXiv. 10.31234/osf.io/qyu69 PMC6509111 31073156

[ref92] Thornton M.A., Weaverdyck M.E., Tamir D.I. (2019). The social brain automatically predicts others' future mental states. Journal of Neuroscience, 39, 140–148. 30389840 10.1523/JNEUROSCI.1431-18.2018 PMC6325264

[ref93] Tennov D. (1979). *Love and Limerence: The Experience of Being in Love*, New York: Stein & Day.

[ref94] Tomasello M. (1999). The human adaptation for culture. Annual Review of Anthropology, 509–529.

[ref95] Uddin L.Q. (2015). Salience processing and insular cortical function and dysfunction. Nature Reviews Neuroscience, 16, 55–61. 25406711 10.1038/nrn3857

[ref96] Van Overwalle F., Baetens K. (2009). Understanding others' actions and goals by mirror and mentalizing systems: a meta-analysis. Neuroimage, 48, 564–584. 19524046 10.1016/j.neuroimage.2009.06.009

[ref97] Vygotsky L. (1978). Interaction between learning and development. Readings on the Development of Children, 23, 34–41.

[ref98] Wang G., Mao L., Ma Y., et al. (2012). Neural representations of close others in collectivistic brains. Social Cognitive and Affective Neuroscience, 7, 222–229. 21382966 10.1093/scan/nsr002 PMC3277373

[ref102] Xu X., Aron A., Brown L., Cao G., Feng T., Weng X. (2011). Reward and motivation systems: a brain mapping study of early-stage intense romantic love in Chinese participants. Human Brain Mapping, 32, 249–257. 21229613 10.1002/hbm.21017 PMC6870433

[ref42] Xu X., Brown L., Aron A., Cao G., Feng T., Acevedo B., Weng X. (2012). Regional brain activity during early-stage intense romantic love predicted relationship outcomes after 40 months: An fMRI assessment. Neuroscience Letters, 526, 33–38. 22902992 10.1016/j.neulet.2012.08.004

[ref103] Yarkoni T. (2009). Big correlations in little studies: Inflated fMRI correlations reflect low statistical power—Commentary on Vul *et al*. (2009). Perspectives on Psychological Science, 4, 294–298. 26158966 10.1111/j.1745-6924.2009.01127.x

[ref104] Younger J., Aron A., Parke S., Chatterjee N., Mackey S. (2010). Viewing pictures of a romantic partner reduces experimental pain: involvement of neural reward systems. PloS One, 5, e13309. 10.1371/journal.pone.0013309[doi] 20967200 PMC2954158

[ref105] Zayas V., Merrill S., Hazan C. (2015). Fooling around and falling in love: The role of sex in adult attachment. In Simpson J., Rholes S., editors. *Attachment Theory and Research: New Directions and Emerging Themes (pp. 68-96)*. New York, NY, US: Guilford Press.

[ref107] Zayas V., Shoda Y., Ayduk O.N. (2002). Personality in context: an interpersonal systems perspective. Journal of Personality, 70, 851–900. 12498358 10.1111/1467-6494.05026

[ref108] Zeki S., Romaya J.P. (2010). The brain reaction to viewing faces of opposite- and same-sex romantic partners. PloS One, 5, e15802. 10.1371/journal.pone.0015802[doi] 21209829 PMC3013131

